# Initial Clinical Experience Treating Patients With Lung Cancer on a 6MV-Flattening-Filter-Free O-Ring Linear Accelerator

**DOI:** 10.7759/cureus.10325

**Published:** 2020-09-09

**Authors:** Andrew R Barsky, Hui Lin, Amberly Mendes, Alexandra Dreyfuss, Christopher Wright, Emily J Anstadt, Abigail T Berman, William P Levin, Keith A Cengel, Nathan Anderson, Lei Dong, James M Metz, Taoran Li, Steven Feigenberg

**Affiliations:** 1 Department of Radiation Oncology, Hospital of the University of Pennsylvania, Philadelphia, USA; 2 Department of Radiation Oncology, Memorial Sloan Kettering Cancer Center, New York, USA

**Keywords:** lung cancer, non-small cell lung cancer, small cell lung cancer, radiotherapy, imrt, vmat, flattening filter free, radiation, throughput

## Abstract

Introduction

Modern technologies, like intensity-modulated radiotherapy (IMRT) and volumetric-modulated arc therapy (VMAT), have improved the therapeutic ratio of thoracic radiotherapy (TRT) for lung cancer (LC). Halcyon™ (Varian Medical Systems, Palo Alto, CA, USA), a novel 6MV-flattening-filter-free O-ring linear accelerator (6X-FFF ORL), was designed to deliver IMRT and VMAT with greater speed than a C-arm linac. Herein, we report our initial clinical experience treating patients with LC on this linac.

Methods

All patients who received TRT for LC on the 6X-FFF ORL at our institution were retrospectively identified. Patients’ clinicopathologic data, radiotherapy details, early disease-control and toxicity outcomes, dosimetric data, couch corrections, and treatment times are reported.

Results

Between 10/2018-12/2019, 30 consecutive patients (median age 66 years, range 54-94 years) received definitive or post-operative TRT for LC (median 66 Gy/33 fractions; range 5-70 Gy/2-37 fractions) following four-dimensional computed tomography (CT) simulation (97%) using daily kilovoltage KV cone-beam CT (CBCT) (100%) on a 6X-FFF ORL for non-small cell LC (84%) or small cell LC (16%), with 53% receiving VMAT, 43% receiving static-field IMRT, and 77% receiving concurrent systemic therapy. All plans were approved through institutional peer review. The average three-dimensional vector couch correction based on CBCT guidance was 0.90 ± 0.50 cm. The average beam-on and beam on plus CBCT times were 1.7 ± 1.1 min, and 5.0 ± 3.2 min, respectively. Grade 3 dyspnea and fatigue occurred in 3% and 3% of patients, respectively. There were no grade ≥4 toxicities.

Conclusion

In this first clinical report of TRT for LC on a 6X-FFF ORL, daily CBCT-guided treatment was fast and safe with respect to dosimetry and clinical outcomes. Thus, use of this linac for TRT may increase LC patient throughput without a detriment in radiotherapy quality.

## Introduction

Clinical management of primary lung cancer (LC) is guided by pathologic evaluation, staging, comorbidities, multidisciplinary discussion, and shared decision-making with the patient [1,2]. Patients who present with early-stage disease [3,4], locally-advanced disease [5-7], distant metastatic disease [8,9], or even recurrent disease [10], may have a National Comprehensive Cancer Network (NCCN) guideline-supported role for thoracic radiotherapy (TRT) as a definitive, neoadjuvant, and/or adjuvant treatment modality, with or without systemic therapy [1, 2].

Technological advances in radiotherapy (RT) delivery have improved the therapeutic ratio of TRT, such that NCCN deems computed tomography (CT)-planned three-dimensional conformal RT (3D-CRT) a minimum technologic standard. NCCN also states that use of more advanced technologies, including intensity-modulated radiotherapy (IMRT) and volumetric-modulated arc therapy (VMAT), is appropriate to deliver RT safely [1]. IMRT and VMAT utilize physician-outlined “contours” of CT-based oncologic target tissues and critical organs-at-risk (OARs) to generate RT dose distributions that maximize target coverage, minimize OAR doses, and optimally, decrease toxicity. IMRT has been shown to reduce high-grade RT pneumonitis and cardiac dose in patients receiving TRT for Stage III non-small cell LC (NSCLC) compared to 3D-CRT, with comparable survival and disease-control, suggesting its role as a standard of care [11]. VMAT, a subtype of IMRT, is delivered in dynamic, continuous arcs that allow for reduced RT treatment times compared to static-field IMRT [12]. Further, static-field IMRT and VMAT may be administered using a flattening-filter-free (FFF) linear accelerator (linac), which can increase dose rate, and decrease head scatter and penumbra in comparison to flattening-filtered (FF) RT [13].

Halcyon™ (Varian Medical Systems, Palo Alto, CA, USA), a novel 6 megavolt (MV)-flattening-filter-free (6X-FFF) O-ring gantry linac, was built to administer RT with greater speed and throughput than a C-arm linac (CAL), with some similarities to TomoTherapy (Accuray, Sunnyvale, CA, USA) [14]. Published reports of 6X-FFF O-ring linacs (ORL) are largely limited to pre-clinical planning studies, and have demonstrated similar plan quality with faster calculated treatment times for 6X-FFF ORLs versus CALs [14-19]. Reports of the clinical use of a 6X-FFF ORL to date have been limited to breast cancer [20], gynecologic cancers [12], and malignant pleural mesothelioma (MPM) [21], and have demonstrated fast treatment times and high throughput with the 6X-FFF ORL compared to CALs, with comparable plan quality, toxicity, and early disease-control.

In this report, we describe our initial clinical experience treating patients with TRT for LC on a 6X-FFF ORL. We hypothesized that dosimetry and treatment times for TRT on the 6X-FFF ORL would compare well with those of CALs.

## Materials and methods

In this IRB-approved, retrospective analysis, we reviewed all patients that received TRT on a 6X-FFF ORL for LC at our institution between 10/2018 and 12/2019. The only exclusion criterion included receiving one or more fraction of RT on a linac other than the 6X-FFF ORL. Electronic medical records were assessed for clinical, RT planning, treatment timing, and image-guided RT (IGRT) data.

Prescriptions, constraints, and treatment planning

Each patient was computed tomography (CT) simulated, in most cases with four-dimensional (4D)-CT, in the supine position, with a knee-foot lock and arm shuttle. A positron-emission tomography (PET)/CT, compression belt, and Vac-Lok™ bag (CIVCO Radiotherapy, Orange City, IA, USA) were used at the discretion of the treating physician.

For patients with intact disease or gross postoperative recurrence, a gross tumor volume (GTV) was contoured to encompass gross parenchymal and/or nodal disease identified on CT and/or PET, when applicable. An internal GTV (iGTV) was generated based upon all phases of the 4D-CT, to account for respiratory motion. The iGTV was uniformly expanded by 0.5-0.8 cm for parenchymal disease and 0.3-0.8 cm for nodal disease, while cropping out of uninvolved organs-at-risk (OARs), like heart, bone, and esophagus, to an internal target volume (ITV). Elective nodal volumes were not included. The ITV was uniformly expanded by 0.5 cm to a planning target volume (PTV). Patients were typically treated to 60-70 Gray (Gy) in 2 Gy fractions. In select cases, where hypofractionated courses were felt to be beneficial in order to expedite RT completion, patients were prescribed 30 Gy, 45 Gy, 55 Gy, and 60 Gy, in 10, 15, 22, and 15 fractions, respectively. For patients receiving postoperative RT (PORT) for mediastinal nodal disease, targets were contoured as per the Lung ART protocol [22], and were treated to 50 Gy in 25 fractions. 

Treatment planning was completed in Eclipse™ (Varian Medical Systems, version 15.6) using 6MV FFF photon static-field IMRT or VMAT. The choice of static-field IMRT versus VMAT was at the discretion of the treating physician, and took into account patient-specific anatomy, as well as the ability to reduce low-dose radiation to the uninvolved lungs. Target coverage goals included that the dose received by 95% of the PTV (D95%) be at least 95% of the prescription dose, and the PTV dose not exceed 110% of the prescription dose. OAR constraints included volume of lungs-iGTV receiving ≥20 Gy (V20) <25% (max<35%), lungs-iGTV mean<18 Gy (max mean<20 Gy), esophagus V60<17%, esophagus mean<34 Gy, heart dose receiving 0.03cc (D0.03cc)<prescription dose, heart mean<20 Gy, spinal cord D0.03cc<45 Gy, and unilateral brachial plexus D0.03cc<66 Gy.

Single isocenter plans were generated for all patients. Planning used three to five arcs for VMAT, or four to six static fields or opposed static fields for IMRT, all with 6X-FFF energy and 800 monitor unit (MU)/minute dose rate. A dual-layer, 1.0 cm wide, stacked and staggered multi-leaf collimator (MLC) system was used for beam-modulation. Beam arrangement varied by technique. For all treatment plans, Photon Optimizer™ was used for optimization and the Analytical Anisotropic Algorithm (AAA) version 15.6 was used for dose calculation [23].

Image guidance

**Figure 1:**
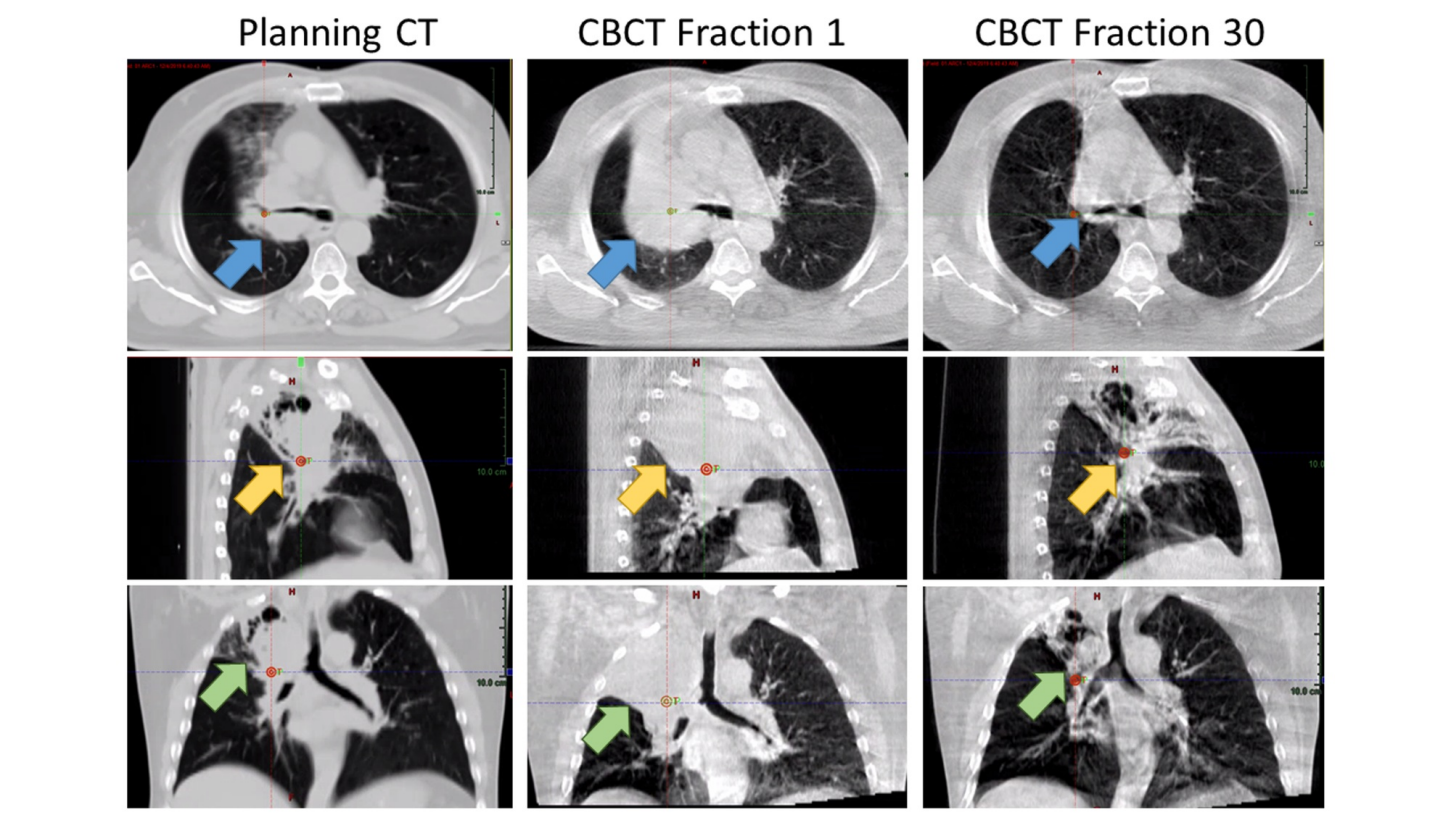
Representative computed tomography (CT) simulation and kilovoltage cone-beam CT (CBCT) images Representative computed tomography (CT) simulation images and corresponding 6MV flattening-filter-free O-ring linear accelerator (6X-FFF ORL) kV cone-beam CT images showing tumor regression in the axial (blue arrows), sagittal (yellow arrows), and coronal planes (green arrows) during thoracic radiotherapy.

All patients received daily IGRT utilizing a kilovoltage (kV) cone-beam CT (CBCT), as the 6X-FFF ORL does not perform kV planar imaging or have an optical distance indicator. Representative CT simulation and kV CBCT images showing tumor regression during TRT are shown in Figure 1.

Data analysis

Our primary aim was to describe the initial clinical evaluation of the use of a 6X-FFF ORL to deliver TRT to patients with LC, with respect to patient characteristics, treatment procedures, initial disease control, toxicity, dosimetry, couch corrections, and treatment speed. Disease-control was reported as clinical and/or radiographic local, regional, and distant control, and mortality. Common terminology criteria for adverse events (CTCAE) version 5.0 grading from weekly on-treatment and routine follow-up visits was used for acute toxicity. Plan dosimetry was assessed by target and OAR dose-volume histogram (DVH) review. Couch corrections, using daily CBCT match to bony anatomy, carina, or spinal cord from initial simulation, were calculated as the mean vector value for all TRT fractions for each patient. Treatment duration was assessed using the average beam-on time, treatment time (daily CBCT plus beam-on time), and total in-room time for each patient.

Statistics

Statistical analysis consisted of descriptive statistics (means, medians, ranges, standard deviations for continuous variables, and percentages for categorical variables). Couch correction and treatment speed data were compared qualitatively to reference values. SciPy 1.0 software was utilized for data analysis [24].

## Results

Patient clinico-pathologic and treatment characteristics

We assessed 30 consecutive patients with a total of 31 primary tumors (one patient had bilateral primary tumors). The median age at RT and follow-up interval was 66 years (range, 54-94 years) and 11.9 months (range, 0.3-18.7 months), respectively.

Patients’ LC histologies were NSCLC (84%, n=26) and small-cell LC (SCLC) (16%, n=5). TRT was delivered most commonly for Stage III disease (52%, n=16; Stage IV 16%, n=5; Stage II 16%, n=5; recurrent Stage III (rIII) 13%, n=4; Stage I 3%, n=1, treated fractionated because in a patient with synchronous Stage IIB disease). Patients with Stage IV disease were treated with TRT as local consolidative therapy (80%, n=4) or definitive therapy (20%, n=1). Three patients (10%) received PORT following lobectomy: two (67%) for gross recurrence and one (33%) for pathological N2 disease. Seventeen (57%), six (20%), and five (17%) patients received concurrent and sequential, concurrent only, or sequential only systemic therapy with TRT, respectively. Concurrent systemic therapies consisted of carboplatin/paclitaxel (57%, n=13), cisplatin/etoposide (17%, n=4), carboplatin/pemetrexed (13%, n=3), carboplatin/nab-paclitaxel (4%, n=1), carboplatin/etoposide (4%, n=1), and osimertinib (4%, n=1). Sixteen (53%) and two (7%) received immunotherapy and targeted therapy, respectively, as part of their LC management.

Table 1 summarizes patients’ RT details. All were treated supine (100%, n=30) with VMAT (53%, n=16), static-field IMRT (43%, n=13), or a combination (3%, n=1). Four-dimensional CT simulation was used for all but one patient (97%, n=29), with compression belt (70%, n=21), knee/foot lock and arm shuttle (100%, n=30), and Vac-Lok™ bag (60%, n=18). Primary tumors were most commonly right-sided (70%, n=21) and located in the right upper (37%, n=11) or right lower lobes (30%, n=9). TRT targets were most commonly lung, hilum, and mediastinum (55%, n=17), lung (16%, n=5), lung and hilum (13%, n=4), or hilum and mediastinum (10%, n=3). The median TRT dose delivered was 66 Gy (range, 5-70 Gy), in 33 fractions (range, 2-37). Two patients (7%) were planned to receive 55 Gy in 22 fractions and 70 Gy in 35 fractions, but did not complete their RT courses due to cancer-related mortality (one from hypoxemic respiratory failure due to a large malignant pleural effusion complicated by aspiration pneumonia, and one likely from a pulmonary embolism) following two and seven fractions, respectively. IGRT was daily kV CBCT for all patients (100%, n=30).

**Table 1:** Details of radiotherapy course Abbreviations: Gy, Gray; VMAT, volumetric-modulated arc therapy; 4D-CT, four-dimensional computed tomography; IGRT, image-guided radiotherapy; kV, kilovoltage; CBCT, cone-beam computed tomography; RT, radiotherapy * Values out of number of primary tumors, not patients treated (one patient with bilateral disease/RT) º Two patients were planned to receive 55 Gy in 22 fractions and 70 Gy in 35 fractions, but did not complete their courses due to cancer-related mortality following two and seven fractions, respectively.

Variable	Value (%)
Laterality	
Left	8 (27)
Right	21 (70)
Bilateral	1 (3)
Lobe	
Left upper lobe	4 (13)
Left lower lobe	4 (13)
Right upper lobe	11 (37)
Right middle lobe	1 (3)
Right lower lobe	9 (30)
Left lower lobe and right lower lobe	1 (3)
Target	
Lung	5* (16)
Lung, hilum	4 (13)
Lung, hilum, mediastinum	17 (55)
Lung, hilum, mediastinum, supraclavicular fossa	1 (3)
Hilum, mediastinum	3 (10)
Hilum, mediastinum, bronchial stump	1 (3)
Delivered dose (Gy)	
Median	66.0
Range	5.0° - 70.0
Number of fractions	
Median	33
Range	2° - 37
Modality	
VMAT	16 (53)
Static-field	13 (43)
Both	1 (3)
Motion management	
4D-CT	29 (97)
None	1 (3)
Compression belt	
Yes	21 (70)
None	9 (30)
Immobilization	
Knee/foot lock, arm shuttle	12 (40)
Vac-Lok™ bag, knee/foot lock, arm shuttle	18 (60)
IGRT	
kV CBCT	30 (100)
Systemic therapy	
Concurrent only	6 (20)
Sequential only	5 (17)
Concurrent and sequential	17 (57)
None	2 (7)

Table 2 summarizes dosimetric parameters. Nearly all institutional target coverage and OAR planning constraints were met; all plans were approved through institutional peer review. In the few cases where constraints were not met, the reason was extensive PTV overlap with one or more OAR (either accepting a minor variation in OAR constraint, or less commonly, accepting lesser PTV coverage when overlapping with spinal cord).

**Table 2:** Dosimetric parameters of targets and organs-at-risk Abbreviations: PTV, planning target volume; D95, dose received by 95% of a structure; V110, volume receiving 110% of the prescription dose; IGTV, internal gross tumor volume; V5, volume receiving 5 Gy; Gy, Gray; V20, volume receiving 20 Gy; V60, volume receiving 60 Gy; D0.03cc, dose received by 0.03 cubic centimeters of a structure

Variable	Value (%) (median, range)
PTV	
D95	96.6 (93.5 - 100.5)
V110	0 (0 - 0.3)
Lungs-IGTV	
V5	54.6 (19.9 - 80.1)
V20	26.7 (4.7 - 36.9)
Mean (Gy)	14.9 (5.0 - 19.4)
Esophagus	
V60	2.0 (0 - 45.2)
Mean (Gy)	18.7 (4.3 - 41.6)
Heart	
D0.03cc (Gy)	61.9 (16.5 - 75.6)
Mean (Gy)	10.8 (2.6 - 21.0)
Spinal cord	
D0.03cc (Gy)	36.2 (5.4 - 48.8)
Left brachial plexus	
D0.03cc (Gy)	0 (0 - 10.3)
Right brachial plexus	
D0.03cc (Gy)	0 (0 - 61.6)

Initial disease-control outcomes

There were seven deaths (23%), which occurred at a median of 5.9 months (range, 0.3-13.8 months) from start of RT: five (71%) due to progressive LC (predominantly from distant, systemic failure) and two (29%) due to intercurrent causes (one from infection, and one from pulmonary hypertension). One-year overall survival was 73%. No deaths were RT-related. There were two (6%) local (both in-field parenchyma), five (16%) regional (all mediastinal or hilar lymph nodes), and eight (26%) distant failures. Both local failures occurred in patients who simultaneously experienced regional failures, at 9.0 and 10.9 months from start of RT.

Acute toxicity

Twenty-nine patients were evaluable for acute toxicity. Acute grade 2 toxicities occurring in >15% of patients were fatigue, cough, dysphagia, dermatitis, and esophagitis, in seven (24%), seven (24%), six (21%), six (21%), and five (17%) patients, respectively. Acute grade 3 toxicities were fatigue (3%, n=1) and dyspnea (3%, n=1). There were no grade ≥4 toxicities.

Image-guided radiotherapy couch corrections

**Figure 2:**
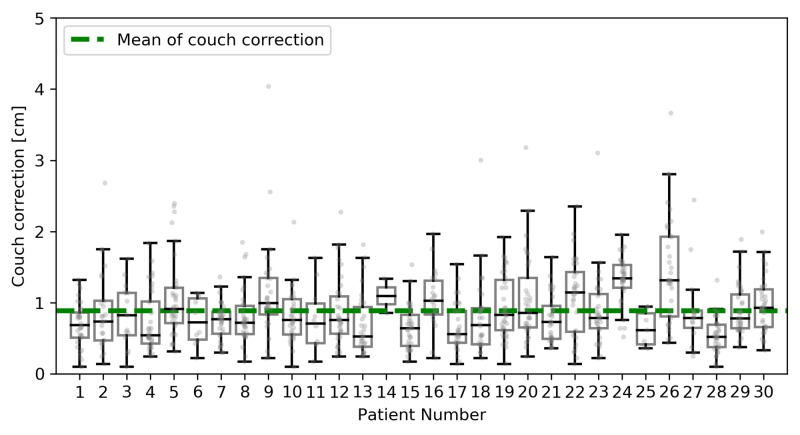
Couch corrections after initial setup based on image-guided radiotherapy Average translational couch correction magnitude using daily kilovoltage cone-beam computed tomography (kV CBCT) for all patients, for all fractions of thoracic radiotherapy.

The average (± one standard deviation) translational couch correction magnitude using daily kV CBCT for all fractions of 6X-FFF ORL TRT for all patients was 0.90 ± 0.50 cm (Figure 2).

Treatment time and throughput analysis

**Figure 3:**
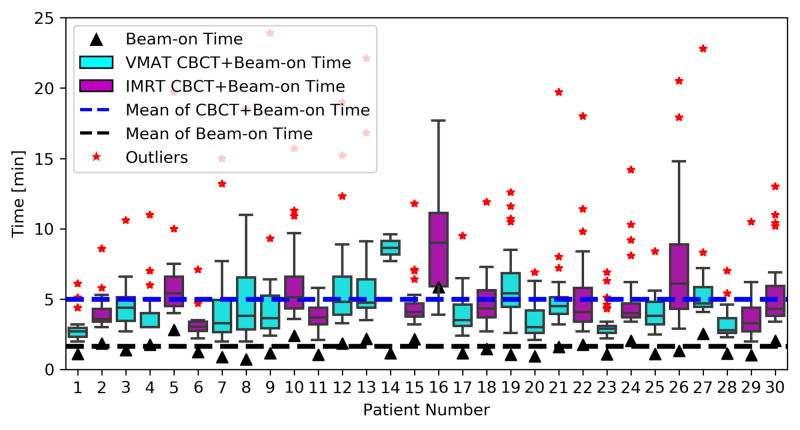
Treatment time Average beam-on and beam-on plus cone-beam computed tomography (CBCT) times for all patients for all non-setup fractions of 6MV-flattening-filter-free O-ring linear accelerator radiotherapy. Treatment times were significantly shorter for patients who received volumetric-modulated arc therapy (VMAT) versus those who received static-field intensity modulated radiation therapy (IMRT) (p<0.05).

**Figure 4:**
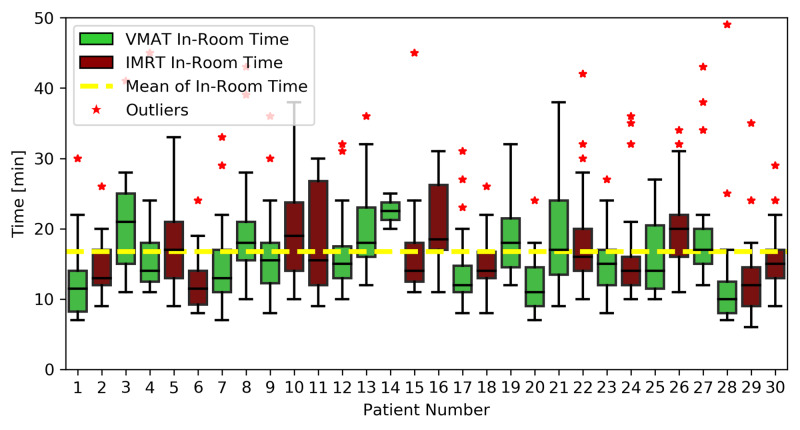
Total in-room time Average total in-room time (time from leaving gowning area to returning to gowning area) for all non-setup fractions of 6MV-flattening-filter-free O-ring linear accelerator radiotherapy. Abbreviations: VMAT, volumetric-modulated arc therapy; IMRT, intensity-modulated radiation therapy

Treatment time is depicted in Figure 3 (beam-on and beam-on plus CBCT times) and Figure 4 (total in-room time) for all non-setup fractions. Outlier times were not excluded from average calculations because the reasons for extended treatment times were not routinely recorded (i.e. excluding quantitative outliers without being certain that they were non-linac-related could bias the results in favor of shorter treatment times if the prolonged times were indeed linac-related). The average beam-on and beam-on plus CBCT times for all patients were 1.7 ± 1.1 min, and 5.0 ± 3.2 min, respectively. The average beam-on and beam-on plus CBCT times for patients treated with VMAT (1.3 ± 0.6 min and 4.6 ± 2.8 min, respectively) were significantly shorter than those for patients treated with static-field IMRT (2.1 ± 1.4 min and 5.5 ± 3.6 min, respectively, p’s <0.05). The average total in-room time (from gowning area to linac back to gowning area) for all patients was 16.8 ± 7.9 min, and did not significantly differ between patients treated with VMAT (16.5 ± 8.6 min) and static-field IMRT (17.0 ± 7.0 min, p=0.39).

## Discussion

Herein, we share the initial clinical experience delivering TRT for patients with LC on a 6X-FFF ORL, which is also the largest published clinical experience of 6X-FFF ORL use to date. We demonstrated the varied clinicopathologic scenarios, initial disease-control outcomes, acute toxicity, dosimetry, couch corrections, and treatment speed of 6X-FFF ORL TRT in LC. The linac was able to deliver TRT to patients with various LC histologies, stages, tumor locations (including bilateral), simulation/setup techniques, and dose-fractionation regimens, indicating that it is capable of delivering IMRT to patients with LC in most clinical scenarios.

We found that TRT planning for LC on a 6X-FFF ORL achieved nearly all institutional dosimetric target and OAR constraints, with the exception of a small number of select, unavoidable circumstances where there was overlap between the PTV and OARs, warranting minor variations in the OAR constraint to maximize target coverage, or PTV coverage to spare spinal cord, at the discretion of the treating physician. All plans were approved via institutional peer review.

IGRT for 6X-FFF ORL TRT with daily CBCT offered acceptable average 3D vector couch correction based on CBCT guidance for all patients (0.89 ± 0.50 cm). This compares well with prior reported average 3D vector couch correction values for TRT for LC, which ranged from 0.53-1.14 cm [25,26]. Yeung et al. found an average 3D vector couch correction of 0.53 cm, calculated by computing the square root of the sum of the squares of the couch corrections utilizing data from daily kV CBCT in each of the left-right, superior-inferior, and anterior-posterior dimensions [25]. Higgins et al. found an average 3D vector couch correction of 1.14 cm, utilizing data from daily CBCT (unspecified whether MV or kV CBCT) with match to carina in patients receiving TRT with parallel opposed fields, 3D-CRT, or IMRT [26]. While statistical comparison between our report and the published studies was not feasible given specific differences between the studies, the couch correction values were qualitatively similar.

The average beam-on, beam-on plus CBCT, and total in-room times for all non-setup fractions were 1.7 ± 1.1 min, 5.0 ± 3.2 min, and 16.8 ± 7.9 min, respectively. The beam-on times observed compared well with previously published beam-on time values for fractionated TRT for NSCLC [27,28]. Mukai et al. reported beam-on times of 4.5 ± 1.3 min and 9.8 ± 1.5 min to deliver IMRT for NSCLC using TomoDirect and TomoHelical systems, respectively [27]. Shrimali et al. reported median beam-on times of 2.6 min and 3.0 min for thoracic VMAT and static-field IMRT, respectively (p=0.03), for NSCLC [28]. Thus, the beam-on times observed for 6X-FFF ORL TRT for NSCLC compared favorably with those of thoracic IMRT on other linacs, even with patients receiving treatment with as many as five arcs in our study. Like Shrimali et al., we observed that beam-on times were significantly shorter for VMAT treatments as compared to static-field IMRT (1.3 ± 0.6 min vs. 2.1 ± 1.4 min, p<0.05), which may be explained by VMAT’s high beam delivery efficiency from dynamic dose delivery and restricted MLC motion per gantry degree, requiring less MU to deliver the same dose as static-field IMRT [28,29]. That said, the total in-room times for patients treated with VMAT vs. static-field IMRT did not statistically differ, which may be a more clinically-meaningful endpoint. As such, clinicians need not avoid static-field IMRT plans out of fears of prolonged patient treatment times relative to VMAT, and should choose the modality that delivers the best plan for the individual patient. At our institution, in situations where high-speed optimization is required, such as online adaptive replanning, static-field IMRT has been the preferred approach, as the ability to deliver IMRT as fast as VMAT allows the focus to remain on plan quality and clinical indication, as opposed to efficiency trade-offs. To the best of our knowledge, no published beam-on plus CBCT or total in-room time data for TRT exist. As a means of comparison, mean beam-on plus CBCT and total in-room times for 6X-FFF ORL breast RT (4.4 ± 0.4 min and 12.4 ± 0.5 min, respectively) [20] and pelvic RT (3.6 ± 0.4 min and 10.8 ± 1.4 min, respectively) are provided [12]. While the total in-room time qualitatively appears longer than the breast and pelvic experiences, it is important to note that those experiences excluded prolonged outlier treatment times that were due to non-linac-related externalities including physician availability for on-board imaging approval, transport staff availability, bladder-filling troubleshooting, and interlock issues requiring physics override. In the pelvic experience, 42 of the 127 time values were excluded for such reasons. In the current experience, outlier times were not excluded, because reasons for prolonged treatments were not recorded. Consequently, we felt that the most conservative way to report the data would be to include all data points, even if it was likely that there were explanations external to the 6X-FFF ORL that accounted for many of the prolonged times.

While prior reports have shown short treatment times and high throughput for patients treated on a 6X-FFF ORL for breast cancer [20], gynecologic cancers [12], and MPM [21], our report is the first clinical report of TRT using this linac for LC, and showed similar results. Quick treatments may improve the patient experience, as treatment tables are often uncomfortable, and maintaining immobility for prolonged periods of time may be difficult, particularly in patients with LC, many of whom may be elderly, frail, and/or have underlying pulmonary and cardiovascular co-morbidities. A high-throughput linac may also help reduce patient wait times and reduce departmental staffing demands.

This report has several limitations. Further follow-up is needed to assess late toxicity and long-term outcomes. As previously discussed, outlier times were not excluded, and therefore our total in-room time values may be longer than the true in-room times for 6X-FFF ORL TRT. This study reports on fractionated TRT for locally-advanced or metastatic LC; stereotactic RT techniques for TRT on a 6X-FFF ORL are beyond the scope of this report, but warrant study. Prospective study of TRT on the 6X-FFF ORL, particularly in comparison to TRT on a C-arm linac, is also a potential direction for future study.

## Conclusions

Herein, we report the initial published clinical experience of 6X-FFF ORL TRT for LC. We showed the linac’s versatility with respect to clinical patient and setup variety, adequacy with respect to dosimetry and couch corrections, and fast treatment times relative to those of CALs. As such, TRT on a 6X-FFF ORL may improve departmental throughput without a detriment in radiotherapy quality.
